# Double Trouble: Massive Unruptured Aortic Aneurysms

**DOI:** 10.5811/cpcem.2017.7.33820

**Published:** 2018-01-09

**Authors:** Martin L. Gagne, Radhika Malhotra, Joseph Zito, Adam Schwartz, Steven Sattler

**Affiliations:** Good Samaritan Hospital Medical Center, Department of Emergency Medicine, West Islip, New York

## Abstract

We describe a patient who presented to the emergency department complaining of generalized weakness, dark stools, and urinary retention who was found to have two large abdominal aortic aneurysms (AAA) compressing his bilateral ureters with associated hydronephrosis and renal insufficiency. In elderly male patients presenting with signs of obstructive uropathy, AAA should be considered as a potential cause.

## INTRODUCTION

Abdominal aortic aneurysms (AAA) can be classified as inflammatory or non-inflammatory. Non-inflammatory AAAs are characterized by asymptomatic presentation, lack of aneurysmal wall thickening, lack of perianeurysmal fibrosis, and normal erythrocyte sedimentation rate. It is known that inflammatory AAAs can result in obstructive uropathy secondary to retroperitoneal/perianeurysmal fibrosis; however, the incidence of non-inflammatory AAAs resulting in bilateral mechanical compression of the ureters is extremely rare. We present a case report of a patient presenting to the emergency department (ED) for generalized weakness, dark stools, and urinary retention who was found to have two large non-inflammatory AAAs causing ureteral compression with bilateral hydronephrosis.

## CASE REPORT

A 77-year-old Caucasian male presented to the ED complaining of generalized weakness, fatigue, lightheadedness, and shortness of breath. His symptoms began gradually three days prior and progressively worsened. His lightheadedness was provoked by moving from a seated to standing position; and at the time he presented, he was unable to stand up. Review of systems (ROS) was positive for decreased urination for one month, as well as dark, maroon-colored stools. ROS was negative for fever, chest pain, nausea, vomiting, and bright red blood per rectum.

His medical history included anemia, blood transfusions, jejunal angiodysplasia, and a cerebrovascular accident. His surgical history was significant for endoscopy, colonoscopy, capsule endoscopy, and deep enteroscopy. Home medications included allopurinol, esomeprazole, ezetimibe-simvastatin, levothyroxine, mometasone, montelukast, and sertraline. In addition, the patient was a former smoker (20 pack-year history) and consumed alcohol occasionally.

Upon presentation to the ED, the patient was afebrile with a heart rate of 80 beats per minute, blood pressure of 104/68 mm Hg, respiratory rate of 24 breaths per minute, and oxygen saturation of 97% on room air. He appeared weak and in mild distress. His body habitus was normal. Mucous membranes were dry. The abdomen was mildly distended, but there was no tenderness, rebound, guarding, organomegaly, or appreciable masses. His rectal exam demonstrated a small amount of dark stool that was guaiac positive. The patient’s skin was pale but warm and dry with good turgor. The remainder of the physical exam was unremarkable.

**Image 1:**
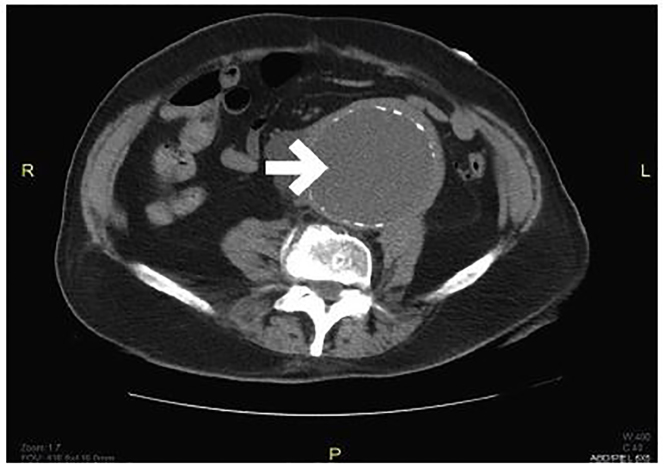
Computed tomography without contrast of the abdomen and pelvis in axial view with an arrow demonstrating a 9 × 9 cm infrarenal aortic aneurysm.

**Image 2:**
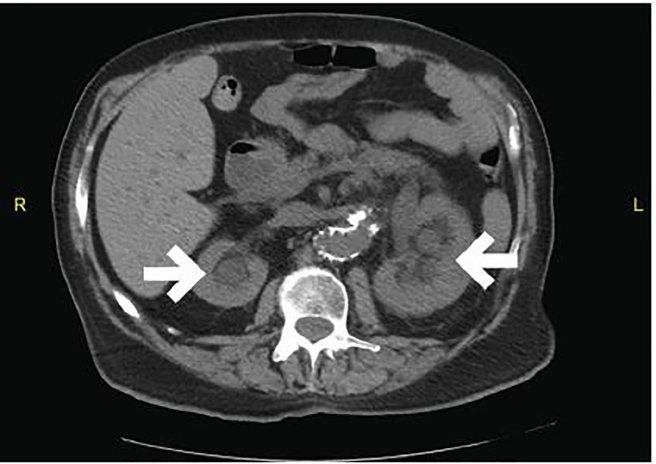
Computed tomography of the abdomen and pelvis in axial view with arrows demonstrating bilateral hydronephrosis

**Image 3:**
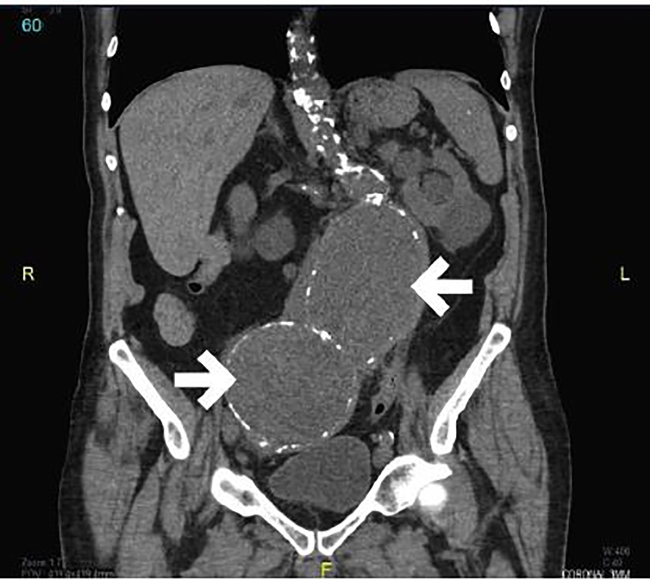
Computed tomography of the abdomen and pelvis in coronal view demonstrates a 9 × 9 cm aortic aneurysm (large arrow) and a 10 × 9 cm right common iliac aneurysm (small arrow)

An electrocardiogram demonstrated a normal sinus rhythm at 79 beats per minute with no acute ischemic changes. Laboratory results were obtained. The patient was found to be severely anemic with a hemoglobin of 5.1 g/dL. His carbon dioxide level was 16 mmol/L, blood urea nitrogen was 104 mg/dL, and creatinine was 3.4 mg/dL. The etiology of the patient’s renal insufficiency was likely due to post-renal obstructive uropathy, as well as pre-renal azotemia related to gastrointestinal bleeding. Two units of packed red blood cells were given. A non-contrast computed tomography (CT) of his abdomen and pelvis was obtained (Images 1–3) to screen for a wide variety of significant intra-abdominal pathologies given the patient’s age, comorbidities, presenting symptoms, and findings suggestive of gastrointestinal bleeding, anemia, and renal insufficiency. The CT revealed two large aneurysms: a 9.0 × 9.0 cm infrarenal abdominal aortic aneurysm and a 10.0 × 9.0 right common iliac artery aneurysm. Both aneurysms were increased in size in comparison to a prior study from 2006. There was no evidence of extravasation; however, this study was performed without intravenous contrast due to the patient’s renal failure. Additionally, there was bilateral ureteral compression from the abdominal aneurysms resulting in moderate bilateral hydroureter and hydronephrosis. There was no evidence of an aortoenteric fistula, perianeurysmal fibrosis, or an acute inflammatory process.

CPC-EM Capsule

What do we already know about this clinical entity?

*Aortic aneurysms are the 14^th^ leading cause of death in the U.S. Common complaints include back, flank, and abdominal pain.*

What makes this presentation of disease reportable?

This patient was found to have two massive aneurysms causing mechanical ureteral compression with bilateral hydronephrosis, which is extraordinarily rare.

What is the major learning point?

In elderly male patients presenting with signs of obstructive uropathy, aortic aneurysms should be considered as a potential cause.

How might this improve emergency medicine practice?

Emergency physicians must continue to consider a wide range of differential diagnoses in elderly patients presenting with generalized or atypical abdominal complaints.

The patient was stabilized and transferred to a tertiary care center for deep enteroscopy, bilateral nephrostomy tube placement, and aneurysmal repair. The patient was later discharged to a rehabilitation center, but soon re-admitted for a recurrence of gastrointestinal bleeding.

## DISCUSSION

Aortic aneurysms were identified as the primary cause of 10,597 deaths in the United States in 2009.^1^ It is the 14^th^ leading cause of death in the U.S.^2^ AAAs are described as a focal dilation, 3 cm or greater, of the abdominal aorta with respect to the original artery. Most AAAs occur distal to the renal arteries.^2^ AAA risk factors include age of 60 years and greater, smoking history (defined as lifetime use greater than 100 cigarettes), hypertension, male gender, and Caucasian ethnicity.^3^ The majority of unruptured aneurysms are asymptomatic and discovered incidentally. The prevalence of symptoms in patients with unruptured AAAs is unknown, but common complaints include back, flank, and abdominal pain. The most common physical exam finding is a pulsatile abdominal mass.

For screening or diagnostic purposes, abdominal ultrasound is the imaging modality of choice (sensitivity of 95% and specificity of 99%); however, CT angiography provides a more detailed assessment of the abdomen preferable for serial monitoring or surgical planning.^2^ CT angiography also has the advantage of being able to identify a ruptured AAA. Surgical management is recommended for patients who have AAAs that are symptomatic or greater than 5.5 cm in diameter. In the hemodynamically stable but symptomatic patient with a large unruptured aneurysm, surgical repair can be delayed for medical optimization with the patient monitored in the intensive care unit setting. Traditionally, AAAs were repaired using an open laparotomy approach; however, technological advances now facilitate an endovascular technique, which is associated with better outcomes.^2^

Between 3%–10% percent of AAAs are characterized as inflammatory AAAs (IAAA), which are a distinct entity distinguished by symptomatic presentation, aneurysmal wall thickening, perianeurysmal fibrosis, adherence to surrounding structures, and an elevated erythrocyte sedimentation rate. IAAAs have a 20–40% incidence of ureteral compromise^4^ and are well documented as causing bilateral obstructive uropathy.^5^–^9^ On the other hand, *non*-inflammatory AAAs have a 0.2% incidence of ureteral involvement^4^ and reports of compressive uropathy related to aneurysmal size are exceedingly rare. Upon review of the literature, we found only two previous case reports of non-inflammatory AAAs causing mechanical ureteral obstruction.^10^,^11^

## CONCLUSION

This case is unique because our patient presented with urinary retention and was found to have renal insufficiency, hydronephrosis, and bilateral ureteral compression from two large aneurysms without preoperative evidence of an inflammatory reaction or fibrotic obstruction. Cases of non-inflammatory AAAs causing mechanical ureteral compression and urinary retention are extraordinarily rare. In elderly male patients presenting with this constellation of symptoms, AAA should be included in the differential diagnosis.
